# Lithium isotopic composition of the carbonate type salt lake in Tibet and its implication for origin and hydrological processes

**DOI:** 10.1038/s41598-025-95913-y

**Published:** 2025-04-07

**Authors:** Jiangdi Zhou, Maoyong He, Binkai Li, Jiangang Jiao, Zhongli Tang, Zhengyan Li, Huihui Rao

**Affiliations:** 1 School of Earth Science and Resources, Chang’an University, Xi’an, 710054 Shaanxi China; 2 State Key Laboratory of Loess Science, Institute of Earth Environment, Chinese Academy of Sciences, Xi’an, 710061 China; 3 Qinghai Provincial Key Laboratory of Exploration Research of Salt Lake Resources in Qaidam Basin. Qaidam Comprehensive Geological and Mineral Exploration Institute of Qinghai Province, Golmud, 816099 China; 4 Qinghai Provincial Key Laboratory of Geology and Environment of Salt Lakes, Qinghai Institute of Salt Lakes, Chinese Academy of Sciences, Xining, 810008 China

**Keywords:** Li-rich salt lake, Lithium isotope, Boron isotope, Source of lithium, Qinghai-Tibetan plateau, Hydrology, Climate-change impacts

## Abstract

The implementation of the carbon peaking and carbon neutrality strategy has led to a steady increase in the supply of lithium resources. Brine is one of the important sources of lithium, and the extraction of Li from carbonate-type brine is particularly straightforward. Research into the source of materials and hydrological processes of brine is crucial for the sustainable development of lithium in carbonate-type brine. As a fluid-mobile and metallogenic element, lithium has a significant mass difference between its stable isotopes (^7^Li and ^6^Li), leading to isotopic fractionation. In this study, we analyzed the hydrochemistry and Li isotope compositions of samples collected from a Li-rich salt lake (Bangor Co) in the Qinghai-Tibetan Plateau. The samples included lake brines, recharge rivers, cold springs, and salt minerals (hydromagnesites). The Li content in the various types of water varied significantly, ranging from 0.06 mg/L to 198.10 mg/L, showing a variation of 4 orders of magnitude. Water samples exhibit a wide range of δ^7^Li values, varying from 4.89‰ to 16.02‰. Notably, the lowest and highest values are observed in cold springs. Additionally, the concentrations and δ^7^Li values in hydromagnesite differ across various relative ages. The hydrochemistry indicated that the recharge water is influenced by rock weathering, but the lake brine is influenced by evaporation concentration. The analysis of trace elements and Li isotopic data reveals that rock weathering, geothermal systems, salt minerals, and freshwater, primarily from early geothermal activities and the redissolution of carbonate minerals, contribute to the Li in salt lake brine. Boron isotopes and lithium isotopes of lake brines are found to vary differently. The δ^7^Li in brine is increased significantly by adsorption of hydromagnesite. And ^11^B gradually accumulates in hydromagnesite. This study has demonstrated that hydromagnesite plays a crucial role in influencing the characteristics of Li in brine.

## Introduction

Lithium (Li) is a key metal used in fields such as batteries, nuclear industry, ceramics, glass, pharmaceuticals, aerospace, etc^1,2^. Particularly in the rapidly developing field of clean energy, Li is a crucial component of rechargeable batteries^3–7^. The demand for it has increased sharply^8^. Currently, Li resources comprise brine types, pegmatite types, and sedimentary types, with brine forms representing 60% of the global identified reserves^9^. Researches concentrate on the Andes Plateau in western South America, the Western Plateau in North America, and the Qinghai-Tibetan Plateau in China. Li resources exploration and production are approximately 57.55 million tons (Li Carbonate Equivalent, LCE) in China, ranking third in the world. Among these resources, brine-type Li resources are mainly produced in the Qinghai-Tibetan Plateau, accounting for 90.9% of its total Li resources^10–12^.

There are multiple sources of lithium in the Li-rich salt lakes. Previous researchers have done a lot of work on the source of lithium, and the exposed rocks on the surface have undergone weathering and erosion, releasing lithium elements from them^13,14^. Initial sediments also include trace of lithium, which are carried into the lake through weathering processes^15,16^. Additionally, deep hydrothermal fluids and shallow geothermal waters can interact with rocks to release lithium components^17–20^. Finally, it is supplied to the salt lake in the form of volcanic jets and hot springs^21^. In addition, the late stage lithium rich magma also brought a large amount of lithium to the salt lake^22^. In summary, the main sources of Li-rich salt lake mineralization can be attributed two main sources: (1) Related to hydrothermal fluids; and (2) weathering of rocks and sediments^12,20,23–32^. However, Orberger^19^ noted out that each salt lake has its own unique geological, hydrological and regional climate history, leading to variations in the source and evolution process of lithium resources may be different. Brine precipitated salt minerals may also serve as a the source of B in salt lakes^33–36^. Therefore, it is worth investigating whether lithium may also originate these sediments. Currently, research on Li-rich salt lakes in the Qinghai-Tibet Plateau primarily focuses on chloride and sulfate types^10,12,25,30–32,37^. Compared to carbonate type Li-rich salt lakes, the first two have higher Mg/Li, which increases the difficulty and cost of lithium extraction from brine^38,39^.

In recent years, with the analytical advancements in isotope geochemistry^40–42^, there has been a growing understanding of the large mass differences between Li isotopes and B isotopes, which lead to large isotope fractionation during geological processes^43,44^. Li and B isotopes have been widely used in the study of salt lakes^12,31,32,35,36,45–47^. However, the changes of Li and B isotopic compositions after the precipitation of calcium carbonate minerals (hydromagnesite) from brine are still controversial, which hinders the in-depth understanding of the characteristics of Li and B isotopic changes in the salt lake.

The Bangor Co Salt Lake is a typical carbonate-type salt lake in the Qinghai-Tibetan Plateau. It is a small-scale source-sink system, replenished solely by a singular surface river (Graza River), characterized by relatively simple influencing elements. Additionally, a significant amount of Li-rich clay minerals and carbonate salt minerals have been deposited at the lake’s bottom and surrounding areas^48^. It is a natural experimental site for studying Li-rich carbonate-type salt lakes.

**Fig. 1:**
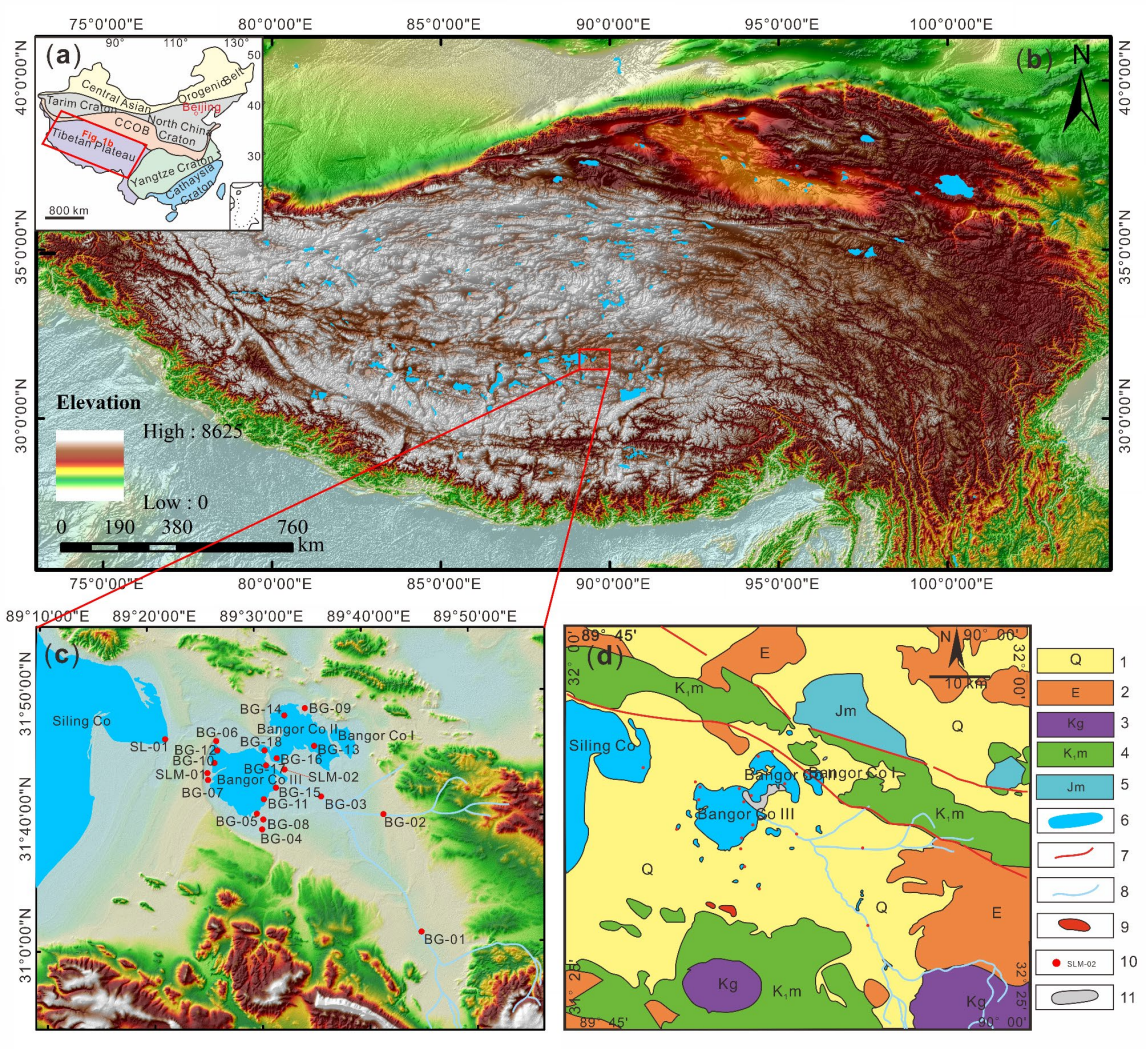
Simplifed tectonic (**a**) and topographic (**b**) maps of China, topographic (**c**) and geological (**d**) maps of the Bangor Co^35^ (1. Quaternary; 2. Tertiary; 3. Cretaceous granite; 4. Cretaceous; 5. Jurassic; 6. Lake; 7. Fault; 8. River; 9. Sinter; 10. Sample location and numbers; 11. Hydromagnesite). This map was processed using ArcMap version 10.8 (https://www.esri.com/).

In this study, various types of water and sediment samples were collected, and their locations were plotted on Fig. 1c. The major cations, trace elements, Li, B concentration, and isotopic composition of Li and B from the samples were analyzed (B isotopes were analyzed by Li^35,49^. The purposes of this study are to: (1) reveal the sources of Li in the research area; (2) explore the influence of carbonate minerals on Li and B isotopes in lake surface brine; and (3) evaluate the impact of lake desalination on brine Li mines. The study will provide a deeper and broader understanding of the genetic mechanisms and influencing factors of brine-type Li deposits in the Qinghai-Tibetan Plateau.

## Geological background

The Bangor Co Salt Lake, which is located in the north of the Qinghai-Tibetan Plateau in China, which is rich in Li resources. It is situated in the east of the Silin Co Lake, which is the largest lake in Tibet^49,50^. The lake surface is approximately 140 km^2^, with an altitude between 4520 m and 4525 m. This area belongs to a plateau arid climate region where the annual accumulated precipitation and annual evaporation are 271.1–495.1 mm and 1740.6–1898.2 mm respectively, with the rainfall mainly concentrating from June to September^51^. Furthermore, the average temperature of the Bangor Co area does not exceed 1 ℃^52^.

**Fig. 2:**
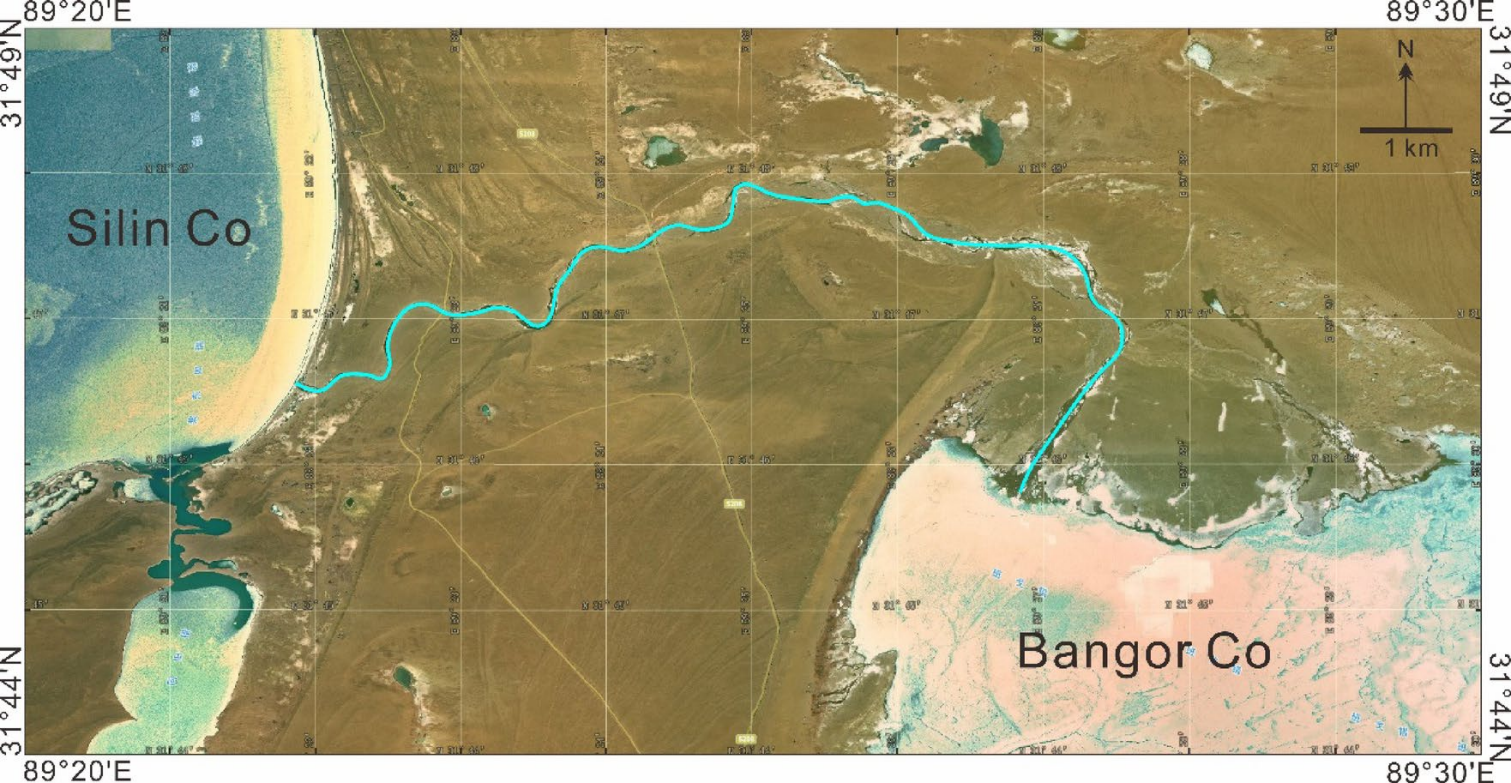
Satellite image map of the Bangor Co area. This map was formed from a screenshot of the SIWEIearth remote sensing satellite (https://siweiearth.com/sw-nav/nav).

Topographically, the Bangor Co Salt Lake is a semi-enclosed basin system between mountains. Notably, it consists of three small lakes, Bangor Co I, Bangor Co II and Bangor Co III (Fig. 1c, d). The Silin Co Lake and the Bangor Co Salt Lake are a unified lake before the late Pleistocene, but later split into two lakes^53^. Earlier, there was much debate about whether there was an exchange of water between the Silin Co Lake and the Bangor Co Salt Lake, but recent satellite images have shown that there is a channel connecting the two lakes (Fig. 2). The Graza River is the only perennial river in the Bangor Co Salt Lake, which flows into the Bangor Co III from southeast to northwest (Fig. 1c). It originated from Langqing mountain and has a length of approximately 180 km^54^. The river freezes in winter, when the flow is small, and the flow increases sharply in summer. In addition, several intermittent rivers develop around the Bangor Co Salt Lake during the rainy season^51^. There are also several cold springs around the Bangor Co Salt Lake, which can feed the lake.

Geologically, the Bangor Co Salt Lake is located in the Bangong–Nujiang Suture Zone (BNSZ) in the central Tibetan (31°43′ N, 89°29′ E) (Fig. 1a, b). It is located in a secondary depression basin within the Silin Co–Lunpola tectonic fault basin^54^. The fault structures in the north edge are well-developed^53^. The base rock in the Bangor Co area consists mainly of the granitic Bangor pluton and the Xueru pluton in the southern part of the lake district^55^. Jurassic-Cretaceous limestones and marlstones, Tertiary sandstones and conglomerates and Quaternary limestones account for the main strata in the study area. The lake basin is made up of sediments from Quaternary and Neogene, consisting of clayey silt, carbonate sinter, borax, and mirabilite^56^. In addition, the Bangor Co area also deposited a large amount of hydromagnesite^57^.

## Materials and methods

### Sample collecting

In the winter of 2015, a total of 21 samples were collected from the Bangor Co area (Fig. 1c). Among these samples, 3 samples were taken from river water, 6 samples were from cold spring water, 1 sample was from the Silin Co Lake, 9 samples were from the Bangor Co lake surface brine, and 2 samples were from carbonate minerals. The pH, density, and temperature of water samples were measured by a portable meter in the field. The details are provided in Table 1.

**Table 1:** Feld sample collection description, and measurement of temperature (t) and density, chemical composition, δ^11^b, and δ^7^li of water samples for the rivers, cold springs, and surface brine.

No.	T	pH	Density	River discharge	Description	Na^+^	K^+^	Ca^2+^	Mg^2+^	Cl^−^	CO_3_^2^^−^	HCO_3_^−^	SO_4_^2^^−^	Li^+^	B^3+^	TDS	δ^11^B	δ^7^Li
	(°C)		(g/cm^3^)	( m³/s )		(mg/L)											(Mean ± 2SD ‰)	
BG-01	7.00	8.40	1.00	1.23	Graza river	34.68	5.33	33.55	19.62	5.97	n.d.^†^	213.75	35.25	0.06	0.81	353.62	4.62 ± 0.25	6.89 ± 0.06
BG-02	9.00	8.40	1.00	0.2	Tributaries of the Graza river	40.30	4.94	35.65	23.25	9.35	n.d.^†^	233.59	49.10	0.09	0.85	400.49	−0.52 ± 0.52	7.67 ± 0.09
BG-03	15.00	8.90	1.00	1.4	Graza river	62.95	10.00	36.68	30.18	18.33	19.51	253.43	68.23	0.13	1.14	504.73	2.93 ± 0.18	7.79 ± 0.10
BG-04	13.00	8.00	1.00	small	Southern spring of Bangor Co	1127.75	174.55	6.45	93.00	841.35	390.20	967.10	1132.75	3.05	20.68	4802.84	−1.26 ± 0.17	12.28 ± 0.03
BG-05	15.00	8.00	1.00	small	Southern spring of Bangor Co	27.40	7.33	56.95	20.00	28.31	n.d.	193.92	70.70	0.05	0.75	439.05	−3.30 ± 0.17	16.02 ± 0.15
BG-06	8.00	8.50	1.00	small	Northern spring of Bangor Co	86.93	22.42	46.30	49.58	36.52	n.d.	555.46	308.00	0.51	5.05	1121.98	7.66 ± 0.19	4.89 ± 0.09
BG-07	16.50	8.10	1.00	small	Southern spring of Bangor Co	59.18	14.84	77.40	68.55	39.84	n.d.	628.86	28.78	0.26	1.04	925.07	−5.96 ± 0.32	5.65 ± 0.18
BG-08	5.00	7.90	0.97	small	Southern spring of Bangor Co	23.22	5.96	37.93	41.13	4.91	n.d.	342.70	20.30	0.08	0.73	487.42	−7.35 ± 0.40	7.67 ± 0.03
BG-09	13.00	8.00	1.00	small	Northern spring of Bangor Co	407.25	45.43	12.15	65.93	255.16	39.51	495.45	442.25	0.93	5.31	1788.99	−0.04 ± 0.19	11.64 ± 0.13
BG-10	14.00	9.20	1.08	n/a^*^	Bangor Co III Lake brine	56290.00	6941.00	25.60	431.00	20016.85	5755.51	6794.47	66540.00	125.20	817.64	165554.23	1.07 ± 0.19	14.84 ± 0.08
BG-11	15.00	9.00	1.09	n/a^*^	Bangor Co III Lake brine	58880.00	6961.00	30.20	440.00	20734.55	6231.07	6943.26	69660.00	125.90	824.23	172662.24	1.29 ± 0.15	14.90 ± 0.02
BG-12	13.00	9.00	1.08	n/a^*^	Bangor Co III Lake brine	58990.00	7137.00	49.30	454.00	20677.83	6096.93	6930.86	68530.00	140.70	822.83	171657.67	1.45 ± 0.25	14.98 ± 0.09
BG-13	14.00	9.00	1.14	n/a^*^	Bangor Co II Lake brine	68050.00	8501.00	32.50	397.00	24778.04	8230.86	9113.03	77570.00	162.80	1028.29	200148.20	3.59 ± 0.26	15.33 ± 0.15
BG-14	13.00	9.20	1.12	n/a^*^	Bangor Co II Lake brine	75080.00	9698.00	22.20	536.00	27495.18	8413.77	9063.43	84330.00	175.40	1052.27	218204.14	1.48 ± 0.26	14.87 ± 0.16
BG-15	16.00	9.10	1.10	n/a^*^	Bangor Co III Lake brine	95140.00	10730.00	37.30	528.00	28438.05	9718.51	8840.26	123600.00	198.10	1113.23	280817.30	0.59 ± 0.17	15.04 ± 0.11
BG-16	13.00	9.10	1.12	n/a^*^	Bangor Co II Lake brine	69030.00	8152.00	373.00	465.00	22259.10	6828.57	7637.58	87390.00	141.30	876.00	205099.11	0.56 ± 0.32	15.14 ± 0.16
BG-17	14.00	9.00	1.11	n/a^*^	Bangor Co III Lake brine	73430.00	9370.00	26.40	523.00	27269.78	8389.38	9819.75	83760.00	180.70	1049.28	216167.06	0.54 ± 0.37	15.31 ± 0.05
BG-18	14.00	9.10	1.12	n/a^*^	Bangor Co III Lake brine	78420.00	7949.00	27.10	459.00	22169.26	6840.76	7674.78	108100.00	147.20	883.79	234634.97	0.38 ± 0.18	15.17 ± 0.19
SLC-01	12.00	8.60	1.00	n/a^*^	Siling Co Lake brine	2680.50	316.95	5.45	158.55	1931.43	510.92	n.d.	3168.00	8.61	57.79	8966.60	0.10 ± 0.22	9.81 ± 0.12
SLM-01	n/a	n/a^*^	n/a^*^	n/a^*^	hydromagnesite (newly-formed)	n.d.^†^	n.d.^†^	n.d.^†^	n.d.^†^	n.d.^†^	n.d.^†^	n.d.^†^	n.d.^†^	100.00	160.00	n/a^*^	n.d.^†^	3.96 ± 0.15
SLM-02	n/a	n/a^*^	n/a^*^	n/a^*^	hydromagnesite	n.d.^†^	n.d.^†^	n.d.^†^	n.d.^†^	n.d.^†^	n.d.^†^	n.d.^†^	n.d.^†^	44.00	100.00	n/a^*^	n.d.^†^	−12.00 ± 0.21
Intercrystalline brine	12.00	9.20	1.16	n/a^*^	Intercrystalline brine	96328.00	17210.00	35.20	528.00	29732.00	9827.00	8840.00	101620.00	192.30	1313.00	293217.40	-3.59±0.15	n.d.^†^

* Note*: Hydrochemistry, δ^11^B and intercrystalline brine data from Li^35^.

*N/A = not applicable.

†N.D. = not determined.

### Elemental determination

All samples were filtered in-situ through 0.45 μm Whatman nylon filters. Each water sample was evenly divided into two parts. A sub-sample for cation analysis was collected in a polyethylene bottle that had been pre-cleaned with secondary distilled HNO_3_ and acidified to a pH of less than 2. The other unacidified sample was collected for anion analysis. HCO_3_^−^ was measured in the field through titration.

The solid sample (hydromagnesite) was cleaned using ethanol and put in an air-dry oven at 40 °C for approximately 10–12 h. Subsequently, it was crushed to 200 mesh with an agate mortar. Next, approximately 2 g of the crushed sample was dissolved in 15 mL of 2% HNO_3_. The solution was then filtered through 0.2 μm Whatman nylon filters and diluted with pure water and 2% HNO_3_ (10‒30 times)^35^. Water samples from the Bangor Co Salt Lake were diluted with 2% HNO_3_ and the concentration of major elements such as Na^+^, K^+^, Ca^2+^, Mg^2+^, Cl^−^, and SO_4_^2−^, as well as trace elements including Li^+^ and B^3+^, were determined. The major ion concentrations (Na^+^, K^+^, Ca^2+^ and Mg^2+^) were measured using inductively coupled plasma optical emission spectroscopy (ICP-OES; iCAP6500 DUO). The Cl^−^ concentrations were tested by ion chromatography (ICS-5000 A). The SO_4_^2−^ concentrations were determined through gravimetric analysis by precipitation BaSO_4_. And the trace elements Li^+^ and B^3+^ were measured using inductively coupled plasma mass spectrometry (ICP-MS; Thermo Finnigan Element II). All analyses for major and trace ions in this study were conducted at the Qinghai Institute of Salt Lakes (ISL), Chinese Academy of Sciences, following their analysis procedure by He^10^.

### Isotopes determination

All lithium and boron isotopes pretreatments were conducted in a class 100 laminar flow fume hood within a class 1,000 clean laboratory at the State Key Laboratory of Loess Science, Institute of Earth Environment, Chinese Academy of Sciences (SKLLQG, IEECAS).

#### Lithium chemical purification procedure

The lithium separation procedure follows the steps described by He^58^. Each water sample containing 300 ng Li is dried in a Teflon^®^ beaker, and the dried sample is dissolved in 0.5 M HNO_3_ (2 mL). The process of purifying Li involves the use of cation exchange microcolumns. These microcolumns are 25 cm in length and have an inner diameter of 6.4 mm. They are filled with 8 ml of resin (Bio-Rad^®^ AG 50 W-X12, 100–200 mesh). The resin was first cleaned and regenerated using 6 M HNO_3_ (32 mL) and Milli-Q ultra-pure water (8 mL). Subsequently, the internal environment of the microcolumn was adjusted with 0.5 M HNO_3_ (12 mL). The sample, which was dissolved in 0.5 M HNO_3_ (2 mL), was then loaded onto the resin. Finally, it was eluted with 0.5 M HNO_3_ (18 mL), and 31 mL of the fraction containing Li was collected. During this process, 4 mL of eluent collected before and after the Li elution peak was separated to evaluate whether the sample was completely leached of Li. To ensure that the collection of pure Li solutions has a Na/Li ratio of less than 1, two procedures are required for samples with high Na/Li ratios such as seawater or lake salt water, due to the close elution peaks of Li and sodium. Na does not affect the measurement of Li isotope ratios.

The entire pre-treatment and purification procedures involve the use of highly purified acid obtained through two rounds of distillation using the Savillex DST-1000 sub-boiling distillation system. Additionally, a 0.5 M ultra-pure HNO_3_ was titrated. The δ^7^Li values of GBW-Li, NASS-6, and AGV-2 were determined to be 8.21‰, 30.88 ± 0.08‰, and 6.18 ± 0.08‰. According to Li^12^, Gou^59,60^ and He^61^, the recommended values are 8.33‰ ± 0.20‰, 31.08 ± 1.24‰, and 6.83 ± 0.75‰, respectively. These results suggest that the purification process did not lead to any significant fractionation. Furthermore, the blank sample introduced to monitor the background process was analyzed using ICP-MS, revealing a lithium concentration of 0.001 ppb, which is negligible compared to the 300 ng (approximately 10 ppb) present in other samples. This suggests that the sample remained unaffected by environmental factors during processing.

#### Analysis of Li and B isotopes

The pure Li solution was evaporated and dissolved in 2% HNO_3_ before being analyzed by the multi-collector inductively coupled plasma mass spectrometer (MC-ICP-MS, Neptune Plus). The testing method was based on the procedures outline in Li^12^ and He^58,61^. External calibration (L-SVEC) was performed during the testing process using the standard bracketing calibration method (SSB) as described by Tomascak^62^. The L-SVEC provided by NIST^63^ was used as the reference standard in this SSB study. The recommended value for the ^7^Li/^6^Li ratio to correct instrument drift during the analysis process is 12.1025 ± 0.0016. We test each sample three times to obtain three measurement values through standard calibration, and calculate their mean and standard deviation. Use the following calculation method for standard correction:

δ^7^Li = [(^7^Li/^6^Li)_Sample_/ (^7^Li/^6^Li)_L−SVEC_) – 1] × 1000‰.

USTCL is used as the internal standard (Standard value is − 19.28 ± 0.18‰; 2 s.d., *n* = 26; Tested value in IEECAS is − 19.31 ± 0.12‰; 2 s.d., *n* = 8) and SPEX-Li (Long term tested value in IEECAS is 12.22 ± 0.07‰; 2 s.d., *n* = 35)^59,60^. This experiment measured the δ^7^Li of the standards, aligning with previously published results^12,31,64–68^. And it met the precision requirements of the laboratory for δ^7^Li testing ( ≤ ± 0.5‰)^69^.

The precise analysis of B isotopes via MC-ICP-MS using SSB method was described in our previous research^35^. Here, an aliquot sample solution of 300 ng B was loaded in a single-step chromatography system for column separation. The long-term external reproducibility of δ^11^B is better than ± 0.5‰ (2SD). Two reference materials including NASS-6 and SRM951a are processed and analyzed alongside other samples to evaluate accuracy and reproducibility. Their δ^11^B values are 39.36 ± 0.29‰ and 0.01 ± 0.32‰ respectively.

## Results

The basic properties (including pH value, temperature, density, etc.), major elements, trace elements and isotopic compositions (δ^11^B and δ^7^Li) of the sample are shown in Table 1.

### The hydrochemical characteristics of the Bangor Co catchment

**Fig. 3:**
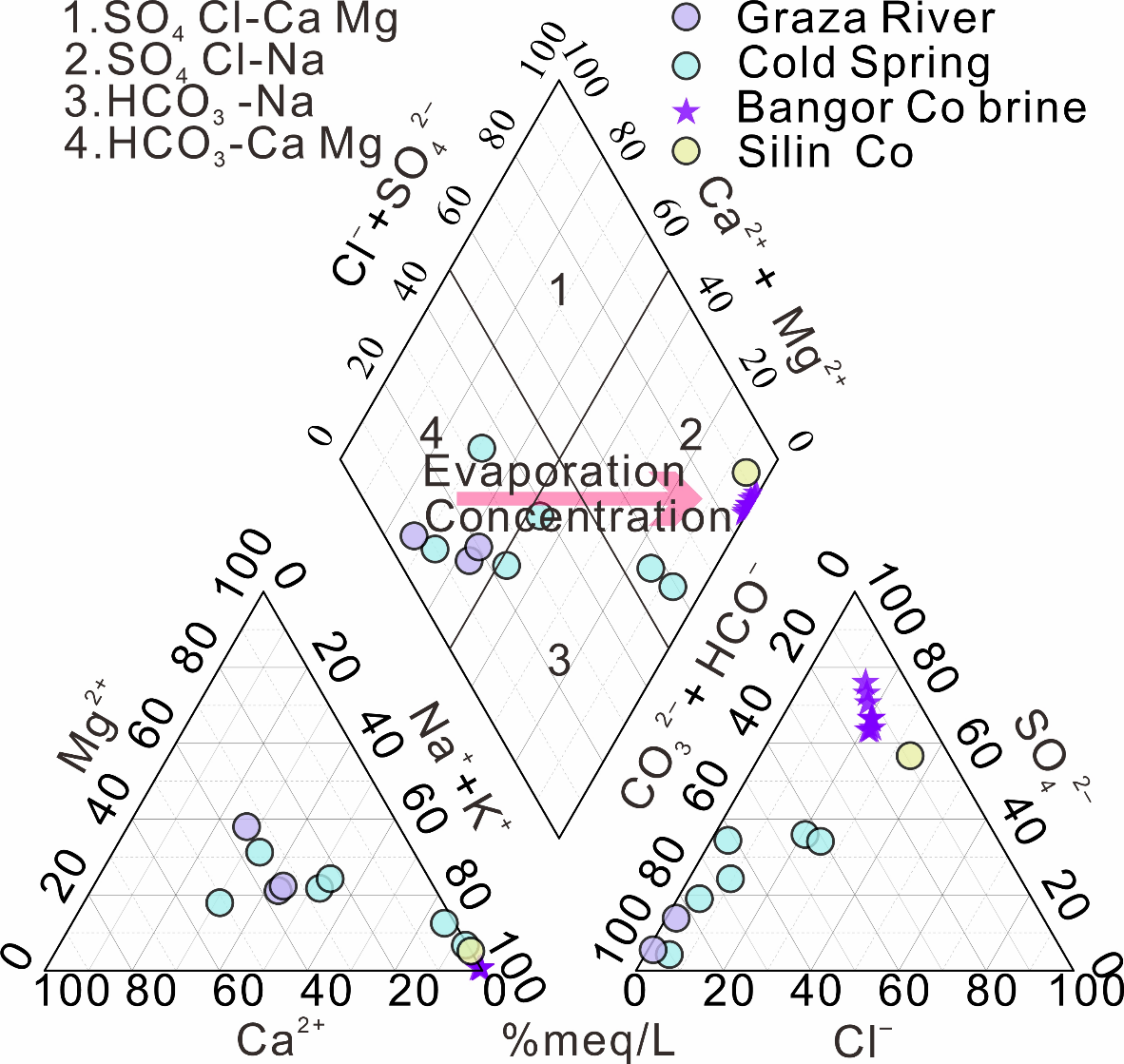
Piper diagram of the relative equivalent proportions of major ions for the water samples of the Bangor Co catchment. (modified from Li^35^).

From Table 1, we can see that pH values in the Bangor Co lake surface brine range from 9.0 to 9.2, which are slightly alkaline. The density of lake brine samples ranges from 1.08 to 1.14. The total dissolved cation and anion (TDS) degree increases to in the following order of river water, spring water, the Silin Co Lake water, and the Bangor Co lake surface brine. In the Bangor Co lake surface brine, the main ion content is ranked as follows: SO_4_^2−^ > Na^+^ > Cl^−^ > K^+^ > CO_3_^2−^ > HCO_3_^−^ > Mg^2+^ > Ca^2+^. The different water samples in the study area were classified into two major hydrogeochemical facies, which SO_4_^2−^ Cl^−^-Na^+^ and HCO_3_^−^- Ca^2+^ Mg^2+^ types (Fig. 3). Li^35^ and Zhou^70^ proposed notable alterations in the hydrochemical properties of the Bangor Co region from the recharge area to the lake area. In the recharge area, rivers and cold spring water are more dominated by rock weathering, with cations relatively balanced and anions biased towards HCO_3_^−^. However, it shifts to evaporative concentration in the lake area, leading to an increasing enrichment of Na^+^ and SO_4_^2−^.

### Li concentration, Li and B isotopic compositions of different samples

The concentrations of Li ([Li]) in carbonate minerals and water samples are listed in Table 1. The average [Li] in river water is 0.09 mg/L, which increases along the direction of the river. However, the lowest value was still significantly higher than the global river average ([Li] = 0.0018 mg/L)^71^. The [Li] in cold spring water near the Bangor Co Salt Lake is slightly higher than that in river water, ranging from 0.05 mg/L to 3.05 mg/L. The high [Li] in BG-04 may be related to early geothermal fluids. As the replenishment water flows into the lake, the water is subjected to intense evaporation and concentration, and the [Li] rises sharply, with an average value of 155.26 mg/L. The [Li] in two hydromagnesites is 100.00 mg/L and 40.00 mg/L. The Li concentration in Silin Co Lake water is 8.61 mg/L.

The δ^7^Li values of Graza River, cold spring, lake brine and hydromagnesite are shown in Table 1, ranging from − 12.00‰ to 15.33‰, and were different from those of the Qaidam Basin Salt Lake (δ^7^Li = 22.69‰)^10^ and seawater (δ^7^Li = 31.0‰)^72^. Additionally, the δ^7^Li of the Bangor Co lake surface brines are higher than those of the recharge water, which is different from the isotopic data of the Kushui Lake and the Lakko Co^12,31,32^.

Specifically, the δ^7^Li values of cold spring ranges from 4.89‰ to 16.02‰. In addition, the δ^7^Li values of new-hydromagnesite and ancient-hydromagnesite are 3.96‰ and − 12.00‰. The δ^7^Li value of the Bangor Co lake surface brine ranges from 14.84‰ to 15.33‰, showing a small fluctuation of ~ 0.46‰ compared to other samples. And the δ^7^Li value of the Silin Co Lake water is 9.81‰.

The concentration of B ([B]) and δ^11^B values reported by Li^35^. All samples have high [B], range from 0.81 mg/L to 1313.00 mg/L. The B isotopic compositions show that in the Graza River, the δ^11^B values range from − 0.52‰ to 4.62‰. Similarly, in cold spring water, the δ^11^B values range from − 7.35‰ to 7.66‰. The δ^11^B values in the brine varied between 0.38‰ and 3.59‰. The two hydromagnesite values are − 1.97‰ and 2.79‰.

## Discussion

### Sources of Li in the Bangor Co salt lake

As good geochemical tracers, Li and B isotopes can identify the characteristics of different fluids^42,73^. To identify the potential sources of Li in the Bangor Co Salt Lake, geochemical tracer methods were used to investigate the relationships between different types of water and solid samples based on elemental proportionality, correlations and Li isotopic compositions. Possible sources of Li resources in the Bangor Co Salt Lake include the weathering of surface rocks, the geothermal system, the redissolution of early salt minerals, and freshwater. A detailed description of each source will be provided below.

#### The impact of rock weathering

**Fig. 4:**
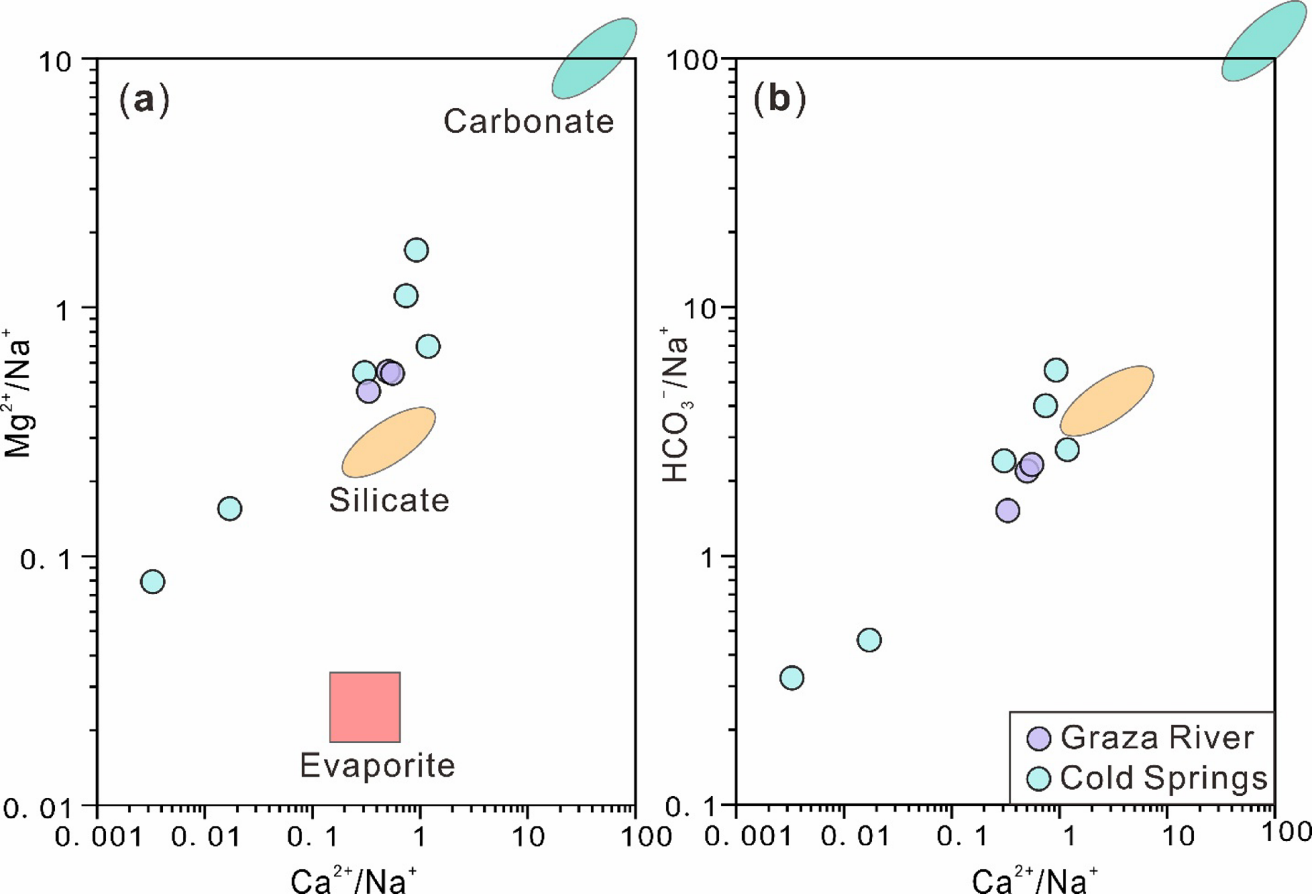
Mixing diagram of the Na-Normalized molar ratios of (**a**) Ca^2+^, Mg^2^^+^ and (**b**) Ca^2+^, HCO_3_^–^ of each recharge water in the Bangor Co catchment. The range data of carbonate, silicate, and evaporite in the mixing diagram were obtained from Gaillardet^71^.

The Silin Co Lake, Graza River and exposed springs in the surrounding area are the main supply sources for the Bangor Co Salt Lake. The source of lithium in Bangor Co Salt Lake is inherently linked to the supply end-members. The relative percentages of major anions and cations in natural water and the distribution of salt minerals in sediments can be used to systematically discuss and evaluate the changes in hydrochemical facies and the sources of major elements in aqueous solutions^45,47,73–77^. Section "The hydrochemical characteristics of the Bangor Co catchment" mentions that recharge water is controlled by rock weathering. The mixing diagram of normalized molar ratios further indicates that silicate rock and evaporite play key roles among them (Fig. 4). In Fig. 4, all the samples located near the silicate (except BG-04, 09) stand out. This indicates that the recharge water in this catchment is dominated by silicate weathering. Cold springs with high total dissolved cation and anion (TDS) will gradually transition to evaporite weathering. Therefore, the source of Li in lake brine is closely related to the weathering of silicate rocks. Nevertheless, the [Li] in river and cold spring is much lower than lake surface brine (Table 1), indicating that rock weathering may not be the primary source of Li element in Bangor Co.

#### Lithium comes from geothermal systems

**Fig. 5:**
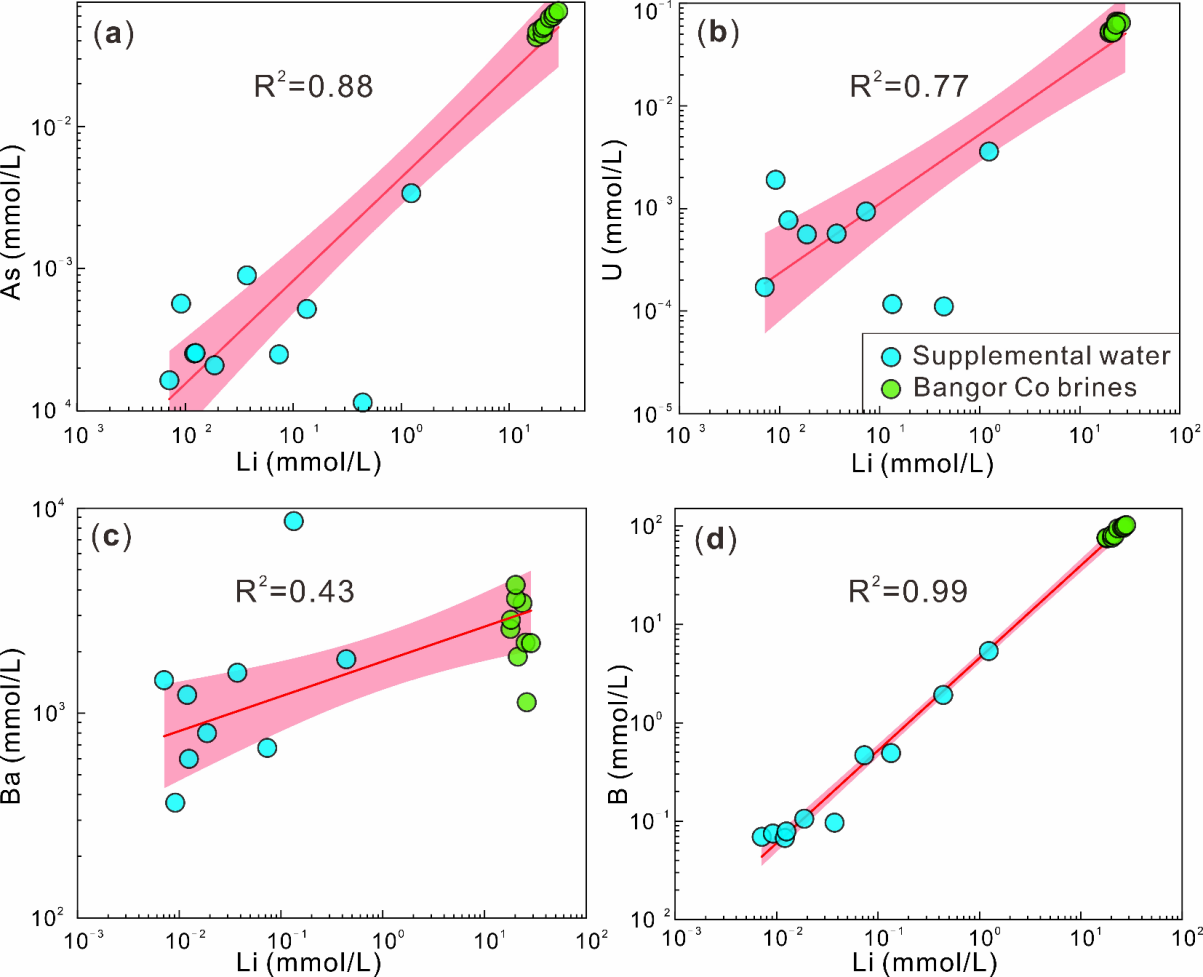
Correlation between Li and As, U, Ba and B molarity concentrations.

**Fig. 6:**
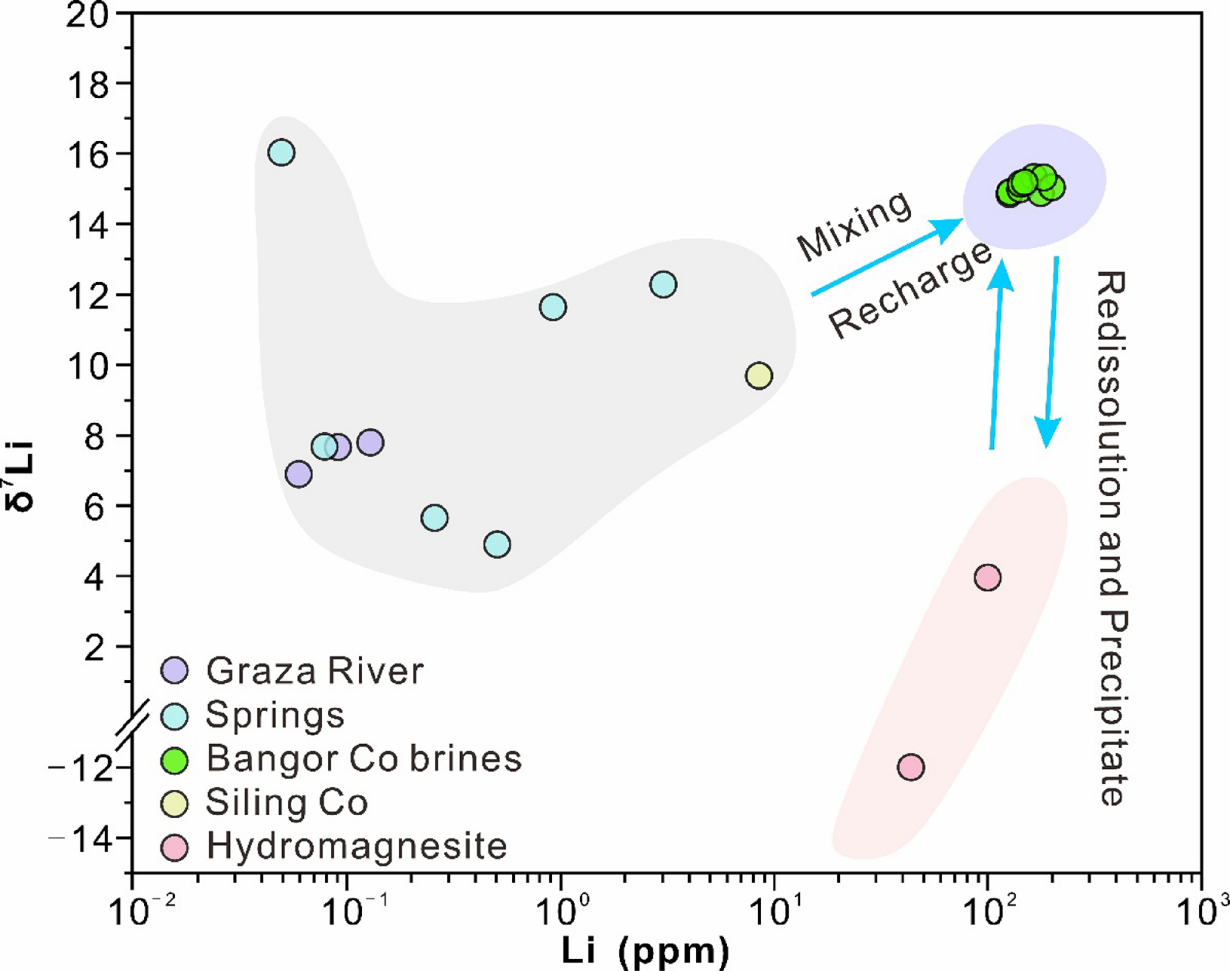
Plot showing the relationship between Li concentrations and δ^7^Li values of brines, rivers, and springs in the Bangor Co catchment and hydromagnesites.

In recent years, previous researchers have conducted extensive studies on Li-rich salt lakes, suggesting that geothermal systems play a significant role in the presence of Li in brine^30,78^. During our field survey, we did not observe any hot springs in the Bangor Co area. However, we did discover a substantial amount of sinter sediments in the southern region of the lake. A significant presence of Li and B-rich hot springs was reported in the Bangor Co area several decades ago^48,79^, and the earliest hot spring activity could be traced back to 34,419 ± 112 a. The geological activity in the area is intensive, with frequent earthquakes^80^, which provides a good migration channel for geothermal fluids. Moreover, Chen^3^ argue that the process of crustal thickening will result in a more significant enrichment of lithium elements. The Qinghai-Tibetan Plateau is characterized by a continental collision setting, where the crust is continuously undergoing thickening^81^. Li is a fluid-mobile element^82^. During the migration of geothermal fluids, water-rock reactions occur with these Li-rich bed rocks (highly fractionated granites) to leach out Li. Therefore, typical deep trace elements and Li concentrations were selected for the correlation analysis (Fig. 5). The results suggest a strong correlation between them, indicating that these elements may share a common source. The δ^7^Li values of geothermal fluids worldwide typically range from − 5‰ to 35‰^83^, and the δ^7^Li values in the Bangor Co area fall within this range (4.89‰ to 15.33‰; Fig. 6; Table 1). The high TDS and [Li] characteristics of BG-04 may be due to its proximity to sinters (Fig. 1d), from which Li is leached into the lake. Additionally, Li^31^ suggest that Li-rich rocks are also an important source of brine Li. The southern part of the Bangor Co Salt Lake contains high levels of [Li], which is found in acidic intrusive rock (~ 100 ppm)^84^. Due to the intense tectonic activity and presence of faults in the area, it is believed that groundwater or hydrothermal fluids undergo water-rock reactions with Li-rich rocks. As a result, Li in the rocks is replenished along the structural faults and transported into the lake area. Based on the existing data, geological background, and previous studies, an early geothermal system may be a source of Li in the Bangor Co lake surface brine.

#### Redissolution of Li from earlier salt sediments

There are many intervals containing salt and salt sediments surrounding the Li-rich salt lake. Because of their low resistance to weathering, it is easy for the Li to be activated and migrate with the fluid^16^. Li^49^ believes that the B in the Bangor Co Salt Lake comes from early clay and carbonate minerals. Li and B have similar sources in salt lakes, so Li may also come from early Li-containing clay and salt minerals. The Bangor Co area is rich in hydromagnesite, which is a common hydrated magnesium carbonate mineral in carbonate-type salt lakes. The area has high [Li] levels of 100.00 mg/L and 44.00 mg/L, as reported by Li^35^. Compare to other samples, the samples in question have light δ^7^Li values of 3.96‰ and − 12‰ (Fig. 6; Table 1). This different also accounts for the high concentration of HCO_3_^−^ in the Bangor Co freshwater.

In addition, in terms of [Li], there is a significant difference in Li content between the two hydromagnesites, which may be due to the formation time. The formation age of the hydromagnesite is differentiated based on the various collection sites. The formation age obtained from the ancient sedimentary cross section is earlier, but the newly-formed hydromagnesite is sourced from the new ore body adjacent to the lake. The Li content of the newly-formed hydromagnesite is significantly higher than that of old hydromagnesite (Table 1). Early-formed minerals are subjected to longer weathering, resulting in more Li being leached out. In summary, early salt sediments can provide Li in salt lakes under the influence of freshwater or circulating brine.

#### Atmospheric precipitation and glacial

Recent research suggests that atmospheric precipitation and glacial meltwater might also contribute some lithium to the salt lakes^10,31^. Wind carries salt into the basin, where it is replenished by precipitation^85^. The ultimate source of freshwater supply in the Bangor Co area is rainfall^86^. We did not collect rainfall data for the region in this field investigation, so we chose atmospheric precipitation data from the Qaidam Basin ([Li] = 1 µg/L, δ^7^Li = 29.4‰)^87^. The δ^7^Li value of rainwater much exceeds that of river water, while the [Li] in rivers is considerably more than that in rainwater, suggesting the incorporation of low δ^7^Li and Li-rich substances during the transit of rainwater to produce rivers. This is far lower than the degree to which secondary minerals influence Li isotope fractionation as previously examined^88^, potentially attributable to shorter rivers (< 80 km). Given that the river at the lake’s inlet has a [Li] of less than 1 mg/L, precipitation alone cannot account for the anomalously high lithium levels in the brine.

Similarly, glacier melting also contributes to the Li in the Bangor Co lake brine. The Bangor Co Salt Lake is not directly supplied by glacier melting but indirectly supplied through Silin Co. Originally, there was no hydraulic connection between the surface of the Bangor Co Salt Lake and the Silin Co Lake, and weak groundwater exchange may have occurred due to the disparity in water levels between the two lakes^56^. Moreover, satellite maps show a channel connecting the two lakes recently (Fig. 2). SLC-01 is the water of the Silin Co Lake ([Li] = 8.61 mg/L). Although its Li content is high, its contribution to Bangor Co is small considering the hydraulic connection of the two lakes.

### The geochemical behavior of Li and B isotopes

**Fig. 7:**
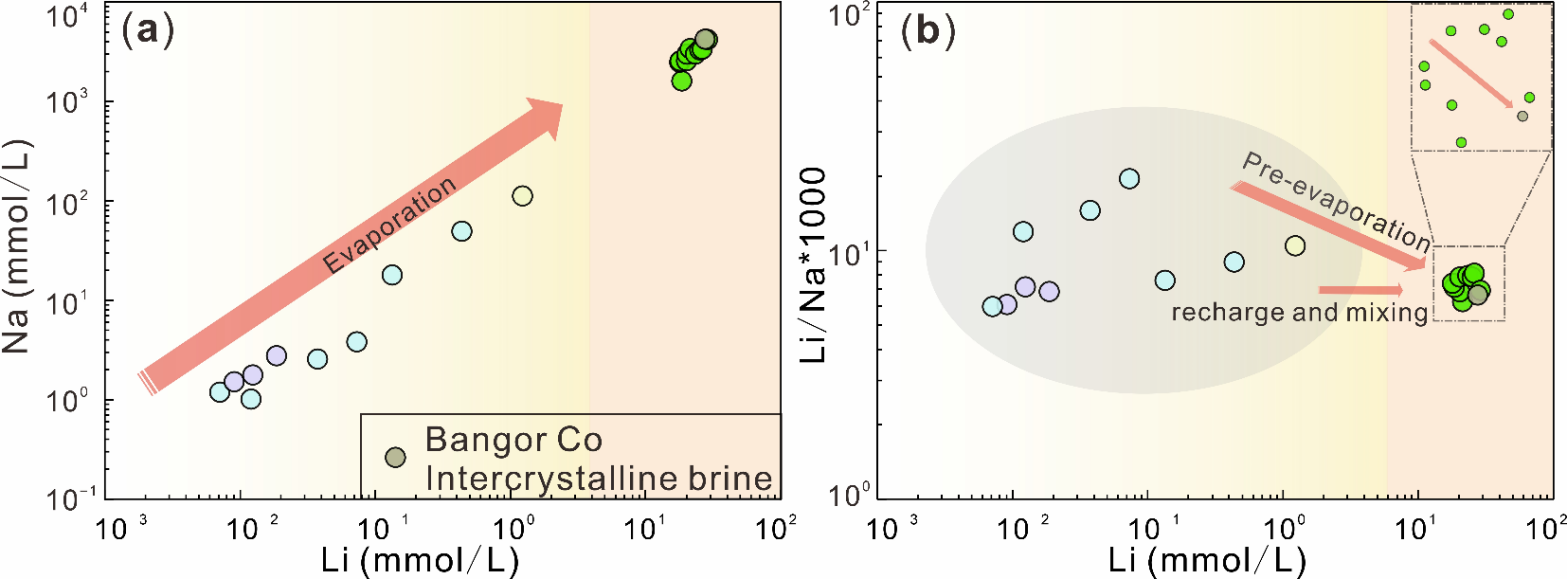
(**a**) and (**b**) plot respectively showing the relationship between Li and Na molarities and the relationship between Li molarities and Li/Na of brines, rivers, springs and intercrystalline brine in the Bangor Co catchment. References to other water sample data are shown in Table 1.

**Fig. 8:**
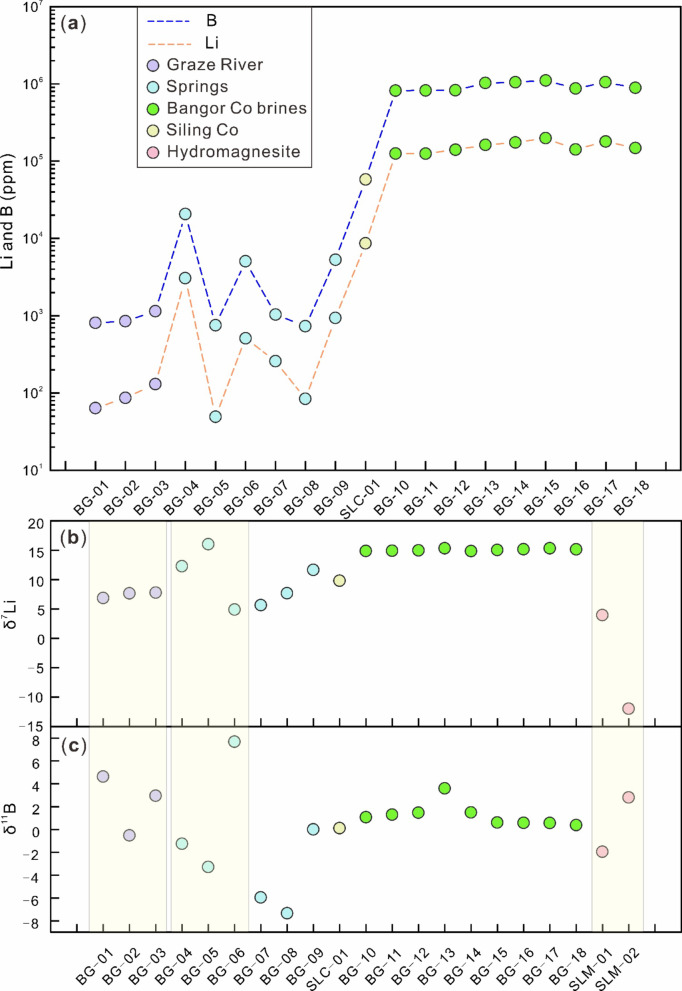
[Li] and [B] of each sampling point (**a**), δ^7^Li features (**b**) and δ^11^B features (**c**).

The movement of rivers and springs can lead to substantial alterations in water composition due to intense evaporation and mixing. The geochemical behavior and source of Li can be inverted to some extent using changes in the Li/Na ratio and its content^89,90^. The [Na] and [Li] in the replenishment water to the Bangor Co lake surface brine show a linear increasing trend due to weathering and evaporation (Fig. 7a) Additionally, [Li] and [B] also exhibit the same trend at each sampling point (Fig. 8a), reflecting that they may have the same potential source. The lake brine samples are all newly formed brine collected along the lake perimeter^35^. Therefore, it remains in the pre-evaporation phase, facilitating the adsorption of secondary minerals and the amalgamation and evaporation of various water types, leading to a marginal reduction in the Li/Na ratio in the lake water (Fig. 7b). Figure 8b and c also show the mixing of lake brine with high and low values of Li and B isotopes in the supply water. Additionally, within the brine, the Li/Na ratio slightly decreases from surface brine to intercrystalline brine, as shown in Fig. 7b. This may be caused by two reasons: First, Li^+^ is replaced into the crystal lattice of carbonate minerals; secondly, intercrystalline brine is mixed with low [Li] water.

Isotopes in the Graza River exhibit a slight increase in δ^7^Li values from the upstream to the downstream, primarily due to the adsorption of secondary minerals^45,91–94^ (Fig. 6). This change is minimal, only 1‰. Despite being a small watershed river, the Graza River still shows preferential absorption of ^6^Li by secondary minerals^31^ (Fig. 8b; Table 1), which significant limit the extent of fractionation. The δ^11^B values decreased because the δ^11^B value of BG-03 decreased due to the mixing of BG-01 and BG-02 (4.62‰, − 0.52‰ and 2.93‰; Fig. 8c; Table 1); Li^35^ consider that the low δ^11^B values of lake surface brine are due to the significant contribution of springs with low δ^11^B values (> 60%), which have lower δ^7^Li values (BG-07 and BG-08, 5.65‰ and 7.67‰) and cannot explain the high δ^7^Li values of lake surface brine. This phenomenon can also be concluded based on theoretical calculations. We used the following binary mixing equation to calculate the δ^7^Li value of the lake brine formed by the mixing of rivers and springs:

δ^7^Li_mix_ = (δ^7^Li_river_ × [Li]_river_ × (1 − f) + δ^7^Li_spring_ × [Li] × f)/([Li]_river_ × (1 − f) + [Li]_spring_ × f). (1)

Where f is the mixing ratio of end-member spring; δ^7^Li _river_ and δ^7^Li _spring_ are the δ^7^Li ratios of end-member river and spring, and δ^7^Li_mix_ is the presumed isotope ratio calculated using the predetermined mixing proportions of the two endmembers. The area has less rainfall, therefore we have excluded the impact of atmospheric precipitation.

We were unable to obtain flux data for each end member in the region, so we made assumptions to constrain their theoretical values: The first assumption is based on Li^35^, who suggest that the contribution of spring water with low δ^11^B values accounts for over 60% of the mixture, resulting in a δ^7^Li_mix_ value of approximately 7.44‰ according to formula (1). The second assumption considers the mixing of spring and river water with the highest δ^7^Li values, resulting in a δ^7^Li_mix_ range of 7.45‰ to 16.02‰. The third assumption involves a normal theoretical calculation, taking the average value of each end member, resulting in a δ^7^Li_mix_ range of 7.45‰ to 9.69‰. It was observed that only the second assumption resulted in a contribution value of 100% from spring water, aligning with the actual lake brine δ^7^Li value. However, this scenario is deemed impossible. Therefore, it can be verified that there are other factors that cause high δ^7^Li values in lake brine.

This may be due to the concentration of ^7^Li in brine during evaporation, and the preferential entry of ^6^Li into clays and salt minerals, especially hydromagnesite, which is described in detail in the next section. Additionally, Fe oxides also play a key role in the enrichment of δ^7^Li in brines^30^, and goethite is also present in the Bangor Co area, which contributes to the increase of δ^7^Li value in brines.

### δ^7^Li and δ^11^B of hydromagnesite from different ages and its significance

The Bangor Co is a carbonate-type salt lake rich in B and Li. This type of salt lake produces a large amount of carbonate precipitation during the evolution process, significantly influencing the Li and B isotope composition of the lake water in the later period^33,34^.

**Fig. 9:**
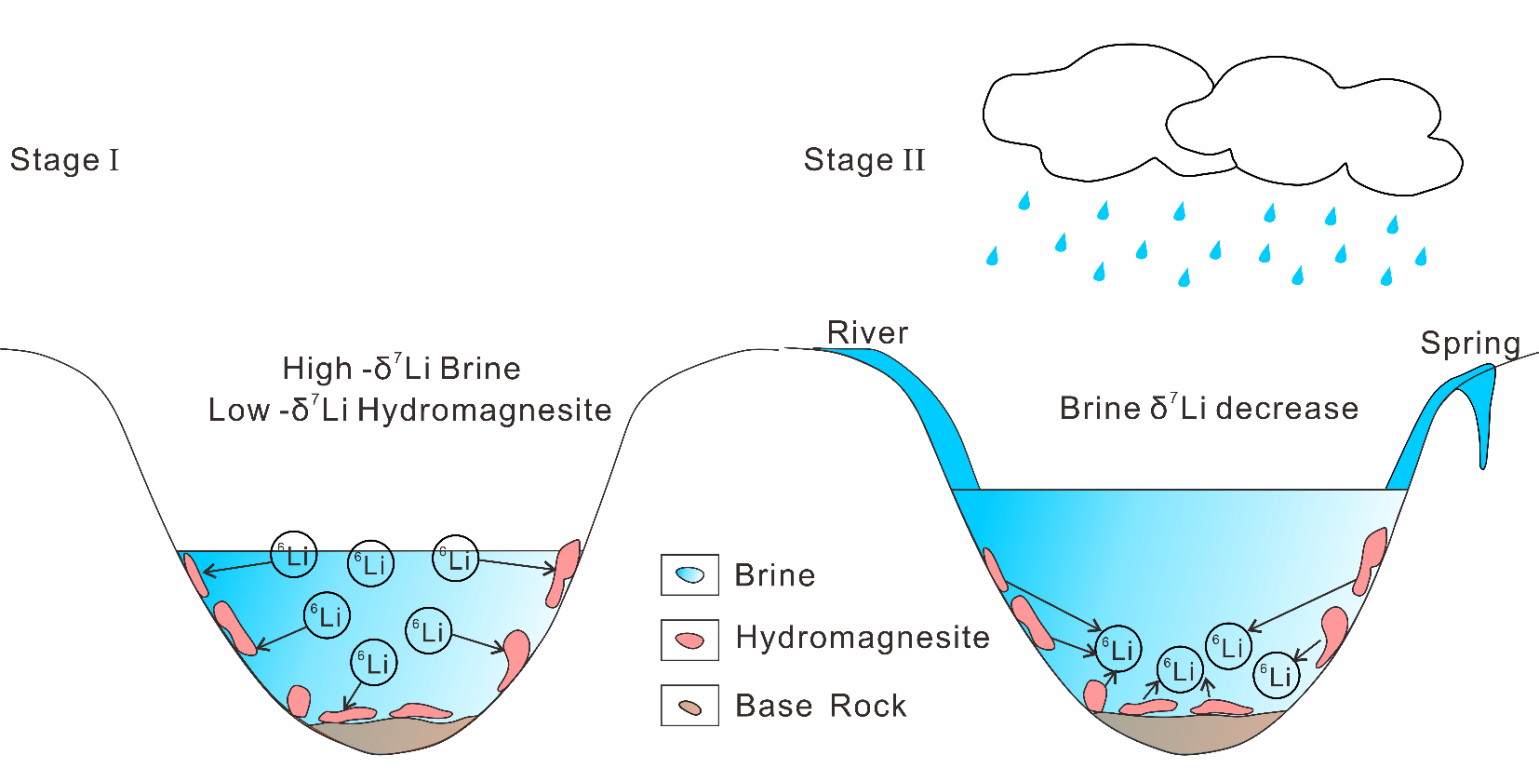
Lithium isotope evolution of brine and hydromagnesite.

The Li isotope fractionation experiment of carbonate precipitation shows that salinity and temperature have little effect on Li isotope fractionation^34^. The precipitation of carbonate minerals such as aragonite and calcite does not change the Li isotope composition of brine^34^, although there is a dearth of evidence on the fractionation of Li isotopes in hydromagnesite. The δ^7^Li values of Bangor Co lake (14.84‰-15.33‰) surface brine are much higher than that of Kuishui Lake (5.94‰-6.35‰)^31^ and Lakko Co (4.97‰-5.25‰)^12^, and similar to those of the salt lake group in southern Qaidam Basin (9.21‰-21.21‰)^10^. The Li-rich salt lakes in Qaidam Basin all have significant fractionation due to large-scale river recharge, resulting in extremely high lithium isotope values in the lake area^30,45^. The small-scale catchment we selected greatly restricted this process, so the δ^7^Li values of Bangor Co should have changed in the lake area. Co-precipitation of carbonate minerals with^6^Li occurs in carbonate-type salt lakes. The receding information indicates that aragonite and calcite have minimal impact on the δ^7^Li values of brines. However, the Li isotopic fractionation of the Bangor Co lake surface brine and hydromagnesite was found to be 27‰ (Table 1), consistent with the Li isotope fractionation experiment on brine salt mineral precipitation conducted by Lin^95^. The high lake surface brine [Li] also eliminates the influence of clay minerals on the δ^7^Li value of the lake surface brine^34^. Li^+^ and Mg^2+^ have a similar lattice and are prone to ion exchange, Li^+^ replaces Mg^2+^ into the mineral lattice, but ^6^Li is easy to enter the solid phase^96^, resulting in the low δ^7^Li value of hydromagnesite. In addition, the δ^7^Li readings of hydromagnesite from various eras exhibit significant variation, paralleling the Li content of hydromagnesite discussed in Sect. 5.2.3. This is attributable to differences in formation time and the adsorption quantity of ^6^Li. Therefore, with the continuous addition of ^6^Li in brine, hydromagnesites have a low δ^7^Li value (Fig. 6). According to the data of this study, the fractionation factor of salt lake brine and hydromagnesite can be calculated as α = 1.0111–1.0274. Based on the above and the B mineralization process^34^, we derived a Li isotope evolution model for hydromagnesite and carbonate-type salt lakes (Fig. 9). The evolution can be divided into two stages: the first stage is the precipitation of hydromagnesite. Due to the substitution of Li^+^ and Mg^2+^ and the easy entry of ^6^Li into the solid phase, the δ^7^Li value of the hydromagnesite is negative, and ^6^Li is enriched more and more in the case of long-term substitution; The second stage is the redissolution of hydromagnesite. The hydromagnesite was dissolved by freshwater and reintroduced into the lake, releasing the previously sequestered ^6^Li, which would lead to a reduction in the δ^7^Li value of the brine. We propose that these two stages may transpire concurrently, with the influx of freshwater and the volume of evaporation being the primary determinants governing this process.

The natural variation of δ^11^B values ranges from − 70‰ to 75‰^97^. This variation is primarily due to the preferential incorporation of 10B into solid phases, such as clay minerals, carbonate co-precipitation, and salt mineral precipitation^98^. Near Lakkor Co, there are numerous carbonate deposits with δ^11^B values significantly lower than those of the surface brine (− 28.08‰ compared to − 4.01‰ to 6.75‰)^36^. This phenomenon is also seen in Damxung Co, where the δ^11^B value of carbonate ranges from − 37.2‰ to − 35.3‰, whereas the δ^11^B value of brine ranges from − 18.5‰ to 17.4‰^33^. The carbonate minerals deposited in the Bangor Co area are mainly hydromagnesite. Boron isotope data show that the difference between hydromagnesite and brine is relatively small (Fig .8c; Table 1). Previous studies suggested that the precipitation of hydromagnesite will not affect the δ^11^B value in brine^34,35^. However, it is worth noting that the δ^11^B value of ancient hydromagnesite is higher than that in brine (Fig .8c; Table 1). There are two possible reasons for this phenomenon: (1) Ancient hydromagnesite experienced longer weathering and strong desorption^10^, B was preferentially desorbed^99^ and (2) When the pH value of the solution is higher, the fractionation direction between brine and sediment changes, and ^11^B preferentially enters the solid phase^100^. In contrast, the non-carbonate type B-rich Li salt lake in Qaidam Basin is in the stage of halite extraction. The δ^11^B value of the brine is similar to that of halite^101^, which does not affect the δ^11^B value.

In summary, the formation and dissolution of hydromagnesite in brine have different effects on B and Li isotopes.

### The impact of lake water desalination on Lithium

In recent decades, global warming has led to glacier melting, increased precipitation, and an expansion of lake areas in the Qinghai-Tibetan Plateau, resulting in significant changes in the content of chemical components in lake water. The Bangor Co is one of the carbonate salt lakes most affected by climate change in Tibet^102^. The area of Vangor Co Salt Lake has increased significantly (59.34–119.48 km^2^)^103^ over the past 40 years from 1976 to 2020. Li^51^ also predict that the water level of Bangor Co Salt Lake will increase significantly by 2024. In 1976, TDS and [Li] in Bangor Co III lake surface brine TDS and [Li] were 221.9 g/L and 127.0 mg/L^54^. In this study, they were found to be 165.3 g/L to 172.7 g/L and 125.2 mg/L to 140.7 mg/L (Table 1). The decrease in TDS means that the lake surface brine is being diluted by fresh water, so the [Li] in it should also be lower, but in fact remains basically unchanged. This suggest that the mixture of brine and fresh water is increasing the solubility of minerals, causing Li elements to migrate into the water. However, on a long-term scale, the Li in the early formed minerals gets consumed. As a result, he TDS and [Li] in the lake water will continue to decrease. The Bangor Co II intercrystalline brine collected in 1976 (TDS and [Li] = 173.5 g/L and 245.0 mg/L) and 2015 (TDS and [Li] = 293.2 g/L and 192.3 mg/L) can well reflect this phenomenon. The storage location and formation time of intercrystalline brine are limited by factors such as reverse weathering, and can only represent the relationship between brine and freshwater. In addition, Silin Co and Bangor Co are already connected by surface waterways, which may gradually form a large lake in the future. Although the concentration of Silin Co Lake water is higher, its volume of water is much larger compared to Bangor Co, which will inevitably result in a reduction of Li levels. In summary, although the freshwater supply to the Bangor Co Salt Lake continues to increase, the redissolution of hydromagnesite results in a period of [Li] increase in the brine, followed by gradual lake water desalination after consuming the hydromagnesite, leading to a decrease in the grade of ore-forming elements.

## Conclusions

This study investigated and analyzed the hydrochemical composition, and the element concentrations, as well as δ^7^Li and δ^11^B of the Bangor Co area. The main conclusions are as follows:

1. The hydrochemical data show that silicate weathering dominates the supply of Li in the recharge area, while in the lake area, it transitions to evaporation and concentration.
2. Lithium in the Bangor Co Salt Lake is a multifaceted coupling source, mainly contributed by geothermal system, salt deposit, and surface rock weathering.
3. The geochemical behavior of Li and B isotopes is different. The ^7^Li in brine increases all the time. And ^11^B gradually accumulates in hydromagnesite. Furthermore, Li isotopes can still undergo fractionation due to secondary minerals in small watershed rivers, although the impact is minimal.
4. In the context of global warming, the precipitation in the Bangor Co area has increased while the evaporation has decreased, and the lake area continues to rise. The Li content in brine will first increase due to the redissolution of Li-rich carbonate minerals. Secondly, when the freshwater supply exceeds the redissolution of salt minerals, the Li content begins to be diluted, affecting the grade of Li resources.

The aforementioned findings offer novel perspectives on the mechanism of source transport and aggregation in brine-type Li deposits, thereby enhancing the feasibility of isotope systems in the field of mineral exploration. It also provides valuable insights for the exploitation of Li resources in salt lakes.

## Data Availability

All data generated or analysed during this study are included in this published article.
